# Unraveling the Intricacies of Curiosity: A Comprehensive Study of Its Measures in the Chinese Context

**DOI:** 10.1002/pchj.813

**Published:** 2024-11-20

**Authors:** Yan Tian, Qi Huang, Xianqing Liu, Jiamin Zhang, Yanghua Ye, Haiyan Wu

**Affiliations:** ^1^ Centre for Cognitive and Brain Sciences and Department of Psychology University of Macau Taipa China; ^2^ State Key Laboratory of Cognitive Neuroscience and Learning IDG/McGovern Institute for Brain Research, Beijing Normal University Beijing China; ^3^ Center for Brain Disorders and Cognitive Sciences Shenzhen University Shenzhen China

**Keywords:** Chinese‐version curiosity scales, confirmatory factor analysis, curiosity assessment, scale reliability, social curiosity

## Abstract

Curiosity, as the strong desire to acquire new information, plays a crucial role in human behaviors. While recent research has delved into the effects, behavioral manifestations, and neural underpinnings of curiosity, the absence of standardized assessment tools for measuring curiosity may hinder advancements in this field. Here, we translated different curiosity scales into Chinese and tested each translated scale by examining its reliability and structural validity. Our results showed that the scores derived from these scales have comparable reliability to those original versions. The confirmatory factor analysis results of the curiosity scales were consistent with previous results. We also found significant associations between different types of curiosity within taxonomy and demonstrated that personality traits such as impulsive sensation seeking, intolerance of uncertainty, and openness can jointly predict trait curiosity. Additionally, we confirmed the social dimension of curiosity, showing that loneliness partially mediates the relationship between social anxiety and social curiosity. This study provides validated Chinese versions of curiosity scales and elucidates the mechanisms of curiosity from multiple perspectives, potentially advancing curiosity research in the Chinese and cross‐cultural contexts.

## 1 Introduction

Curiosity is a desire for knowledge or information coupled with positive emotions, heightened arousal, or a tendency for exploration (Grossnickle 2016), which is perceived as both a trait that varies among individuals (Litman and Spielberger 2003) and an emotional or motivational state that profoundly influences learning and memory processes (Engel 2011; Grossnickle 2016; Klahr, Zimmerman, and Jirout 2011; Oudeyer, Gottlieb, and Lopes 2016; Renninger and Hidi 2015; Von Stumm, Hell, and Chamorro‐Premuzic 2011). Curiosity encompassed both the desire for immediate information and the pursuit of long‐term knowledge (Gottlieb et al. 2013). Throughout the scientific studies, curiosity has been defined in various ways: as a longing to explore the unknown (James 1899), a thirst for knowledge (Freud 1915), a driving motivation for engaging in problem‐solving behavior without substantial reward (Harlow 1950), a need to comprehend and unravel the external world (Cohen, Stotland, and Wolfe 1955), and a form of internally‐driven information seeking (Loewenstein 1994). Through exploring diverse viewpoints, we aim to unveil the nuanced facets of curiosity and contribute to offering a comprehensive exploration of its measures and characteristics delving into its intricacies.

One challenge in studying curiosity is disentangling it from aspects of definitions, dimensionality, and distinctions, as some cognitive concepts are highly related to or even overlap with curiosity, such as information‐seeking and interest (Daw et al. 2006; van Lieshout, de Lange, and Cools 2019). Previous studies have categorized curiosity into different groups based on various classifications, revealing overlaps among them. A long‐standing criterion of classification is in terms of the nature of the object of curiosity, whether it is an experienced object (e.g., physical curiosity; Dewey 1910), a stimulated response (e.g., perceptual curiosity; Berlyne 1954; Litman and Spielberger 2003), a desire for knowledge/information (e.g., epistemic curiosity; Kang et al. 2009; Litman 2005), or an interest in interpersonal information (e.g., social curiosity; Litman and Pezzo 2007; Renner 2006; Robinson, Demetre, and Litman 2017). To elucidate the categorization of curiosity based on the causes, Berlyne (1954) initially made a distinction between two forms: “epistemic” and “perceptual” curiosity. Subsequently, his following work further differentiated between “diversive” and “specific” curiosity based on the range of the object of curiosity (Berlyne 1960). In addition, some categories differentiate curiosity based on its degree of stability—for example, trait versus state curiosity (Loewenstein 1994). Social curiosity, on the other hand, involves an interest in understanding the behaviors, thoughts, and feelings of others, driving the motivational‐behavioral system to facilitate adaptation to social context (Renner 2006; Dunbar 2004).

Curiosity assessment enables researchers to quantify and analyze individuals' various curiosity levels, offering insights into their knowledge acquisition tendencies and exploratory behaviors (Wagstaff et al. 2021). Depending on different classification criteria, researchers have developed well‐validated scale measures to quantify and illustrate various domains of curiosity. For example, Kashdan et al. (2018) created the Five‐Dimensional Curiosity Scale (5DC) and later updated it to the revised version: Five‐Dimensional Curiosity Scale‐revised (5DCR; Kashdan, Disabato, et al. 2020), providing a validated and comprehensive measure of multidimensional curiosity. Other examples include: the Curiosity and Exploration Inventory (CEI; Kashdan, Rose, and Fincham 2004); the Perceptual Curiosity Scale (PCS; Collins, Litman, and Spielberger 2004); the Epistemic Curiosity Scale (ECS; Litman 2008); the Social Scale (SCS; Renner 2006). In organizational research, some commonly used examples include: the Work‐related Curiosity Scale (WCS; Mussel et al. 2011); the Entrepreneurial Curiosity Scale (EnC; Jeraj and Marič 2013); and the M‐Workplace Curiosity Scale (Kashdan, Goodman, et al. 2020). Continuous refinement of measurement tools is essential for advancing research in this multidimensional field.

**FIGURE 1:**
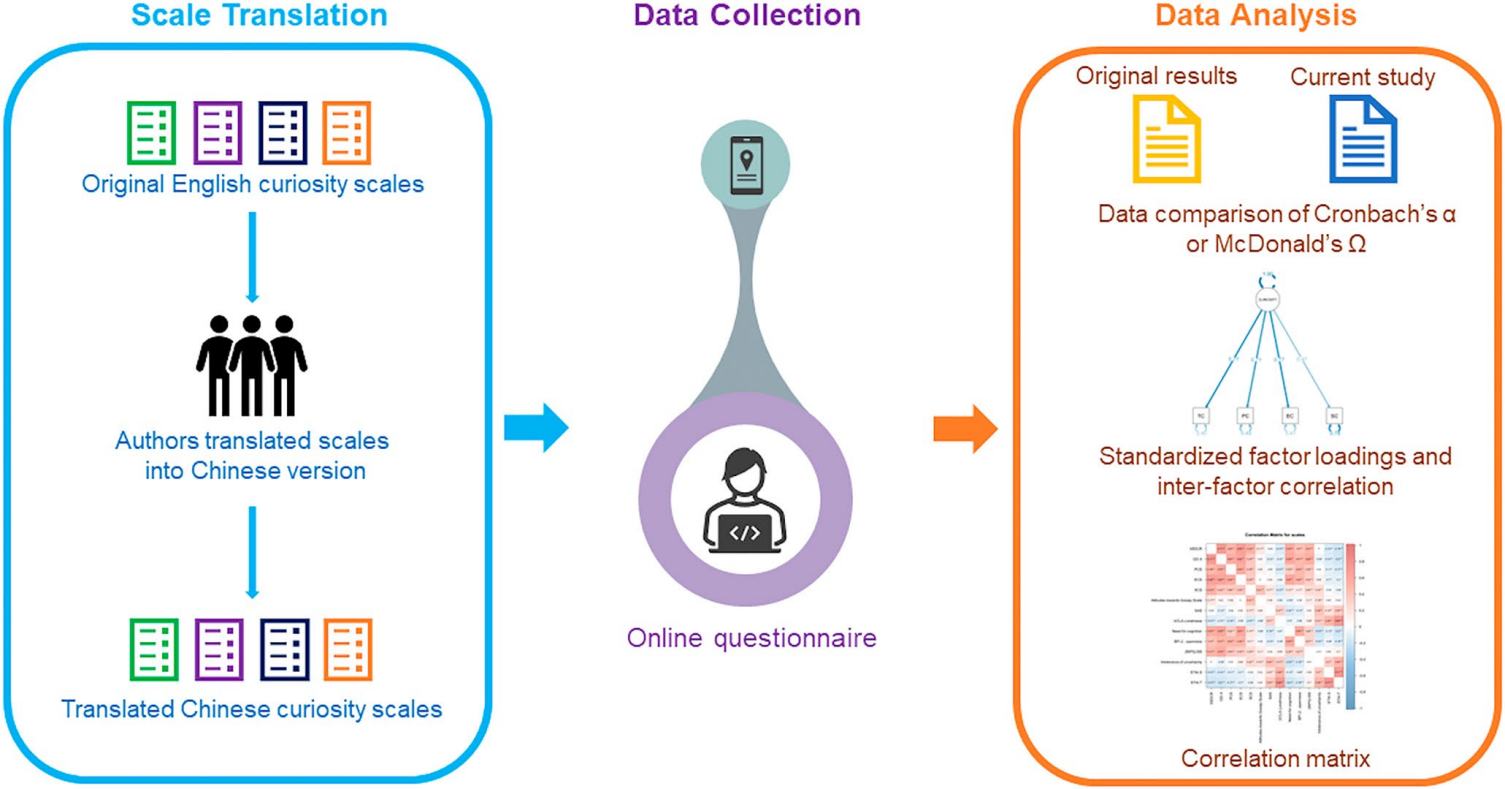
The overview of this study.

Cultural and contextual variations can impact the interpretation of curiosity, leading to challenges in creating universally applicable measures. Prior research has explored curiosity assessment studies in the Chinese context. For example, Ye et al. (2015) validated the Curiosity and Exploration Inventory‐II (CEI‐II), demonstrating the relationship between curiosity, life satisfaction, and human values. Another study focused on the reliability and validity of the Social Curiosity Scale (SCS) among Chinese university students (Zhang 2019). However, these studies have primarily concentrated on singular domains of curiosity and may not thoroughly evaluate the reliability and validity of translated curiosity scales. There is a need to develop correlations among different types of curiosity and explore their interrelationships within an integrative taxonomic framework. In this study, we translated the original English scales and validated their Chinese versions with our sample group. Further, we tried to investigate the relationship between curiosity and other psychological factors (see Figure 1 for the overview of this study). Our findings indicate that there are strong positive correlations among various curiosity dimensions and between curiosity and other psychological factors. Lastly, we discussed how emotional states, such as state anxiety, can influence an individual's desire for novelty and knowledge. This study provides structural evidence of different types of curiosity in the Chinese context through reliability tests, structural validity tests, and correlation analyses across different scales (Figure 1).

## 2 Methods

### 2.1 Scale Translation

All scales were translated into Chinese and proofread by the first five authors of this work (details in Supporting Information). They also checked the original English‐language curiosity scales for any inconsistencies to address potential misunderstandings.

### 2.2 Tests Procedure

This study used an online survey platform (www.wjx.com) and received a total of 831 responses. After quality checks, 723 participants (455 females) were included in the final sample, with ages ranging from 18 to 53 years (*M*_age_ = 22.11, *SD*_age_ = 4.35). Table 1 shows the demographic information. The study was approved by the institutional review board at the University of Macau (BSERE20‐APP011‐ICI), and all participants provided informed consent. The participants received 30 RMB as compensation and had the chance to review their assessment results, which included personality scores and their positions relative to the sample. The researchers anonymized the surveys before submission and embedded additional quality control items in the survey to identify inattentive respondents.

**TABLE 1:** Demographic information.

	Gender	Education						Employment	
	Female	Junior high school	High school	Specialist	Undergraduate	Master	Doctor	Student	Full time employment
Num	455	3	8	28	506	162	16	654	69
%	62.93	0.41	1.11	3.87	69.99	22.41	2.21	90.46	9.54

### 2.3 Measures

We included 13 measures in the study, with 5 curiosity‐related scales (the Five‐Dimensional Curiosity Scale Revised, the Curiosity and Exploration Inventory‐II, the Perceptual Curiosity Scale, the Epistemic Curiosity Scale, and the Social Curiosity Scale) and 8 other scales (the 12‐item Attitudes Toward Gossip Scale, the Social Anxiety Scale, the UCLA Loneliness Scale, Big Five Inventory‐2, the Impulsive Sensation Seeking Scale, the 12‐item Intolerance of Uncertainty Scale, the State–Trait Anxiety Inventory). Each scale's introduction was listed in the Supporting Information.

### 2.4 Statistical Analysis

The study used McDonald's ω for the 5DCR scale, and Cronbach's α was reported for the other scales (detailed in the Section 3). Besides fundamental psychometric analyses, confirmatory factor analysis (CFA) was conducted to validate the multi‐scale structure for each scale. CFA models were fitted in R using the maximum‐likelihood estimation method with the covariance matrix. Model fit was evaluated using the root mean square error of approximation (RMSEA; Steiger 1980), the standardized root mean residual (SRMR; Jöreskog and Sörbom 1996), the comparative fit index (CFI; Bentler 1990), and the Tucker–Lewis index (TLI; Tucker and Lewis 1973). Good model fit was determined if RMSEA/SRMR were ≤ 0.08 and TLI/CFI were ≥ 0.90 (Brown 2006; Hu and Bentler 1999; Klein 2005). Additionally, modification indices (MI) were examined to explore sources of local misfit, and model modifications were only made if justified in substance.

## 3 Results

### 3.1 Descriptive Statistics and Measurement

The score means, standard deviations, and Cronbach's α coefficients of each scale (McDonald's ω coefficient for 5DCR) in our samples were calculated, all scales had high consistency in their internal validity and dimensions (data in Table 2). Generally, all of the Chinese curiosity‐related scales had comparable reliability to the original scales.

**TABLE 2:** Means, SDs, and Cronbach's αs of scales.

Scales	Mean	SD	Cronbach's α in the Chinese sample
5DCR	107.4345	14.89	—
CEI‐II	31.3266	7.04	0.873
PCS	40.3806	8.31	0.869
ECS	26.5362	5.20	0.849
SCS	26.3421	5.81	0.846
ATGS	36.2074	6.69	0.814
SAS	95.7635	19.15	0.932
ULS	46.1093	9.80	0.912
NCS	55.2724	11.21	0.892
BFI‐2‐openness	41.8368	7.82	0.858
ISSS	7.6708	4.30	0.817
IUS	34.6072	9.26	0.896
S‐AI	41.5297	11.29	0.938
T‐AI	45.7234	9.26	0.899

*Note:* 5DCR: Five‐Dimensional Curiosity Scale Revised; ATGS: Attitudes Toward Gossip Scale; BFI‐2: Big Five Inventory‐2; CEI‐II: Curiosity Exploration Inventory‐II; ECS: Epistemic Curiosity Scale; ISSS: Impulsive Sensation Seeking Scale; IUS: The 12‐Item Intolerance of Uncertainty Scale; NCS: Need for Cognition Scale; PCS: Perceptual Curiosity Scale; S‐AI: State Anxiety Inventory; SAQ: Social Anxiety Questionnaire; SCS: Social Curiosity Scale; T‐AI: Trait Anxiety Inventory; ULS: UCLA Loneliness Scale.

#### 3.1.1 Five‐Dimensional Curiosity Scale Revised (5DCR)

**FIGURE 2:**
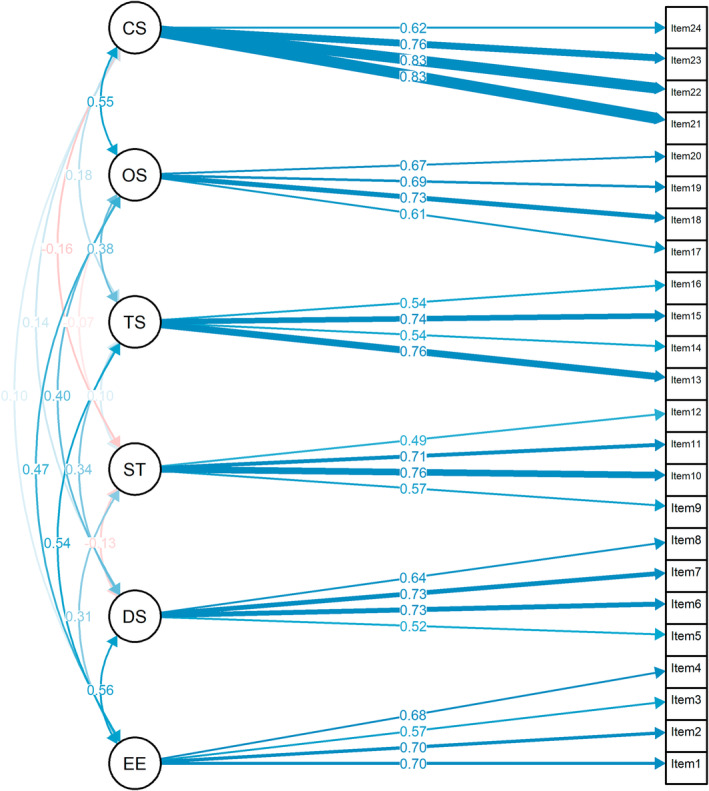
Standardized factor loadings and inter‐factor correlation for the six‐factor 5DCR model for the current sample (*n* = 723). CS: covert social curiosity; DS: deprivation sensitivity; EE: enjoyable exploration (joyous exploration); OS: overt social curiosity; ST: stress tolerance; TS: thrill seeking.

Table 3 shows the statistical results of the 5DCR. The completely standardized results of this model (Figure 2) were consistent with the six‐factor latent structure identified in the previous study (Kashdan, Disabato, et al. 2020). Some model fit indices indicated a good fit for an original model except CFI and TLI (*χ* ^2^ (237, 723) = 848.86, *p* < 0.001; CFI = 0.89; TLI = 0.88; RMSEA = 0.06; SRMR = 0.05). Our results were consistent with those of previous studies (*χ* ^2^ (237, 483) = 888.83, *p* < 0.001; CFI = 0.91; TLI = 0.89; RMSEA = 0.08; SRMR = 0.05, Kashdan, Disabato, et al. 2020), which provided further evidence for the six‐factor structure of the 5DCR. We retained the original six‐factor structure even though modification indices suggested various cross‐loadings and covaried errors, as they tended to be non‐replicable across samples in the original article (Kashdan, Disabato, et al. 2020).

**TABLE 3:** Mean, standard deviations, reliability indices, and correlations among 5DCR measures for the Chinese sample (CS, *n* = 723) and English sample (ES, *n* = 483).

Dimensions	Mean	Deviation	α (Chinese)	ω (Chinese)	ω (English)	Person's correlation				
						1	2	3	4	5
1 JE	20.09	3.84	0.747	0.749	0.870					
2 DS	17.89	4.37	0.740	0.754	0.879	0.42***				
3 ST	14.65	4.56	0.721	0.732	0.882	0.22***	−0.13***			
4 TS	18.98	5.20	0.842	0.842	0.882	0.39***	0.26***	0.04		
5 OS	15.77	4.97	0.740	0.747	0.880	0.34***	0.31***	−0.07	0.29***	
6 CS	20.16	4.25	0.762	0.768	0.869	0.04	0.1*	−0.16***	0.17	0.43***
Total	107.55	15.07	—	—	—	—	—	—	—	—

Abbreviations: CS: covert social curiosity; DS: deprivation sensitivity; JE: joyous exploration; OS: overt social curiosity; ST: stress tolerance; TS: thrill seeking.

* *p*  < 0.05.

** *p*   < 0.01.

*** *p* < 0.001.

#### 3.1.2 Curiosity and Exploration Inventory‐II (CEI‐II)

**FIGURE 3:**
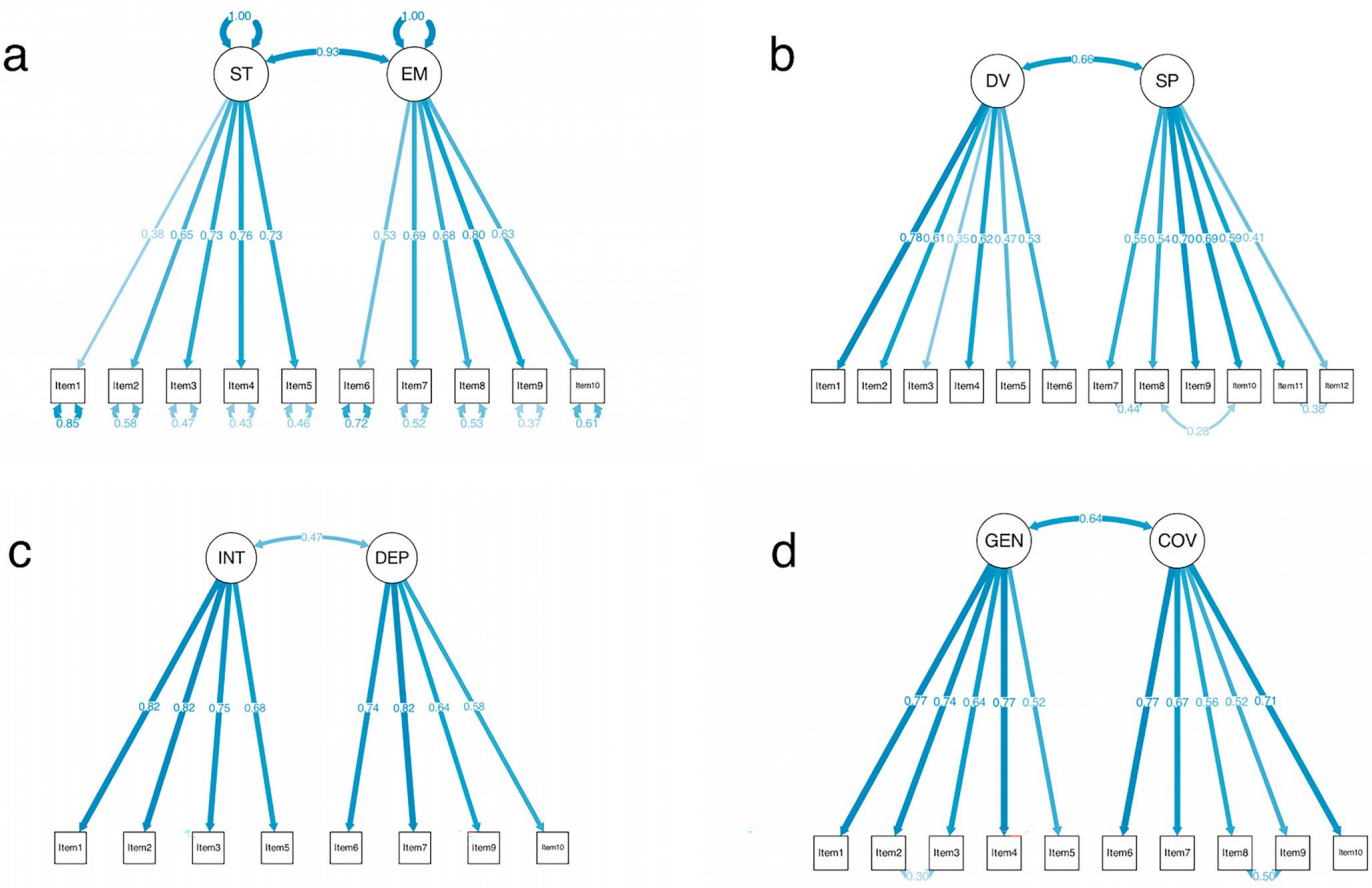
Standardized factor loadings and inter‐factor correlation for the two‐factor models. (a) CEI‐II model; (b) PCS model; (c) ECS model; (d) SCS model for the current sample (*n* = 723). COV: covert; DEP: deprivation; DV: diversive; EM: embracing; GEN: general; INT: interested; SP: specific; ST: stretching.

Table 4 displays the statistical results of the CEI‐II. There is a high consistency in the internal validity of CEI‐II and its dimensions. We then validated the two‐factor (Stretching–Embracing) latent structure identified in the previous samples (Kashdan et al. 2009). The completely standardized results of this model (Figure 3a) were consistent with the previous results (Kashdan et al. 2009). Model fit indices indicated a good fit for an original model (*χ* ^2^ (34, 723) = 176.73, *p* < 0.001; NNFI = 0.93; CFI = 0.95; RMSEA = 0.08; SRMR = 0.04). Our results were consistent with those of previous studies (*χ* ^2^ (34, 150) = 64.62, *p* < 0.01; NNFI = 0.96; CFI = 0.97; RMSEA = 0.08; SRMR = 0.06, study 1 in Kashdan et al. (2009)). The results provided further evidence for the latent structure of the CEI‐II.

**TABLE 4:** Mean, standard deviations, reliability indices, and correlations among CEI‐II measures for the Chinese sample (CS, *n* = 723), and English sample (ES, *n* = 311, study 1 in (Kashdan et al. 2009)).

Dimensions	Mean	Deviation	α (Chinese)	α (English)	Person's correlation
					1
1 Stretching	17.13	3.55	0.786	0.79	
2 Embracing	14.28	4.12	0.798	0.76	0.74***
Total	31.40	7.16	0.877	0.83	—

*** *p* < 0.001.

#### 3.1.3 Perceptual Curiosity Scale (PCS)

Table 5 shows the statistical results of the PCS. The current study had a high consistency in the internal validity of PCS and its dimensions. Model fit indices of the present model indicated a bad fit for the original model (*χ* ^2^ (34, 723) = 461.61, *p* < 0.001; CFI = 0.83; TLI = 0.79; RMSEA = 0.10; SRMR = 0.07). MIs indicated that three pairs of items (Item 11 and Item 12, Item 7 and Item 8, and Item 8 and Item 10) might mutually covary, and the inclusion of the covariance between them improved the model fit. Item 11 “Hear musical instrument/like to see it” and Item 12 “See vocal group/different voice types” described similar curiosity about instruments and chores. Item 8 “Hear strange sound/find out what caused it” and Item 10 “Hear something/see what it is” described similar curiosity about the cause of sound. As the contents of each item pair were closely related based on the proofreading of these items, the three covariance terms were considered reasonable and added to the CFA model. The modified model demonstrated good fit according to most of the fit indices (*χ* ^2^ (50, 723) = 217.78, *p* < 0.001; CFI = 0.93; TLI = 0.91; RMSEA = 0.07; SRMR = 0.05). The completely standardized results of the modified model (Figure 3b) were consistent with the previous results (Collins, Litman, and Spielberger 2004). Subsequent analysis was based on this modified model.

**TABLE 5:** Mean, standard deviations, reliability indices, and correlations among PCS measures for the Chinese sample (CS, *n* = 723), and English sample (ES, *n* = 320).

Dimensions	Mean	Deviation	α (Chinese)	α (English)	Person's correlation	
					1	2
1 Diversive	15.19	3.37	0.72	0.73; 0.78		
2 Specific	15.64	3.79	0.79	0.77; 0.78	0.51***	
3 Unassigned	9.76	2.77	—	—	0.66***	0.54***
Total	40.38	8.31	0.87	0.85; 0.87	—	—

*Note:* The Cronbach's αs in English samples are calculated separately for the male and female samples.

*** *p* < 0.001.

#### 3.1.4 Epistemic Curiosity Scale (ECS)

Table 6 shows the statistical results of the ECS. We found a high consistency in the internal validity of the ECS and its dimensions. We first examined the two‐factor latent structure identified in the sample. Model fit indices indicated a bad fit for an original model (*χ* ^2^ (34, 723) = 364.3, *p* < 0.001; CFI = 0.89; TLI = 0.85; RMSEA = 0.12; SRMR = 0.08; NNFI = 0.88). Item 4 “Enjoy discussing abstract concepts” and Item 8 “Conceptual problems keep me awake thinking” described similar internal motivations for solving conceptual and abstract problems. Item 4 and Item 8 show high residual correlation (MI = 96.25) and may have cross‐loadings (Item 4 to the *Deprivation* dimension, MI = 38.33; Item 8 to the *Interest* dimension, MI =31.2). We deleted Item 4 and Item 8 in the modified model, which then showed an improved fit from the original model (*χ* ^2^ (19, 723) = 148.97, *p* < 0.001; CFI = 0.94; TLI = 0.92; RMSEA = 0.10; SRMR = 0.06; NNFI = 0.92). The completely standardized results of this model (Figure 3c) were consistent with the previous results (Litman 2008). Subsequent analysis was based on this modified model.

**TABLE 6:** Mean, standard deviations, reliability indices, and correlations among ECS measures for the Chinese sample (CS, *n* = 723), and English sample (ES, *n* = 515, study 4 in (Litman 2008)).

Dimensions	Mean	Deviation	α (Chinese)	α (English)	Person's correlation
					1
1 Interest	13.98	3.05	0.83	0.82	
2 Deprivation	12.63	3.02	0.80	0.76	0.51***
Total	26.54	5.20	0.85	—	—

*** *p* < 0.001.

#### 3.1.5 Social Curiosity Scale (SCS)

Table 7 shows the statistical results of the SCS. The current study had a high consistency in the internal validity of SCS and its dimensions. Model fit indices indicated a bad fit for the original model (*χ* ^2^ (34, 723) = 361.84, *p* < 0.001; CFI = 0.88; TLI = 0.84; RMSEA = 0.12; SRMR = 0.07). MIs indicated that two pairs of items (Item 2 and Item 3, MI = 46.49; Item 8 and Item 9, MI = 165.15) might covary, and the inclusion of the covariance between them improved the model fit (*χ* ^2^ (32, 723) = 160.63, *p* < 0.001; CFI = 0.95; TLI = 0.93; RMSEA = 0.08; SRMR = 0.05). As the contents of each item pair (Item 2 and Item 3; Item 8 and Item 9) were quite related based on the proofreading of these items, the two covariance terms were considered reasonable and added to the CFA model. The modified model (Figure 3d) demonstrated a good fit according to most of the fit indices. These results provided further evidence for the validated structure of the scale. The completely standardized results of this model were consistent with the previous results (Renner 2006). Subsequent analysis was based on this modified model.

**TABLE 7:** Mean, standard deviations, reliability indices, and correlations among SCS measures for the Chinese sample (CS, *n* = 723), and English sample (ES, *n* = 311).

Dimensions	Mean	Deviation	α (Chinese)	α (English)	Person's correlation
					1
1 General	14.38	3.18	0.83	0.82	
2 Covert	11.99	3.64	0.81	0.81	0.48***
Total	26.37	5.81	0.85	0.83	—

*** *p* < 0.001.

### 3.2 Mechanisms of Curiosity From the Four Perspectives

After examining the reliability and validity of the curiosity scales, we then tried to comprehend the relationship and the underlying mechanisms of curiosity from different perspectives, including the similarities and differences in the concept of curiosity in taxonomy, curiosity and cognitive science, social development of curiosity, and the interplay between emotion and curiosity. Specifically, we aimed to discuss (1) the relationship between general curiosity (trait curiosity) and diverse curiosity (perceptual curiosity, epistemic curiosity, and social curiosity), (2) the relationship between five‐dimensional curiosity and other curiosity, and (3) how to improve the framework and possible new taxonomy.

#### 3.2.1 The Similarity and Distinction Among Different Curiosity Scales

**FIGURE 4:**
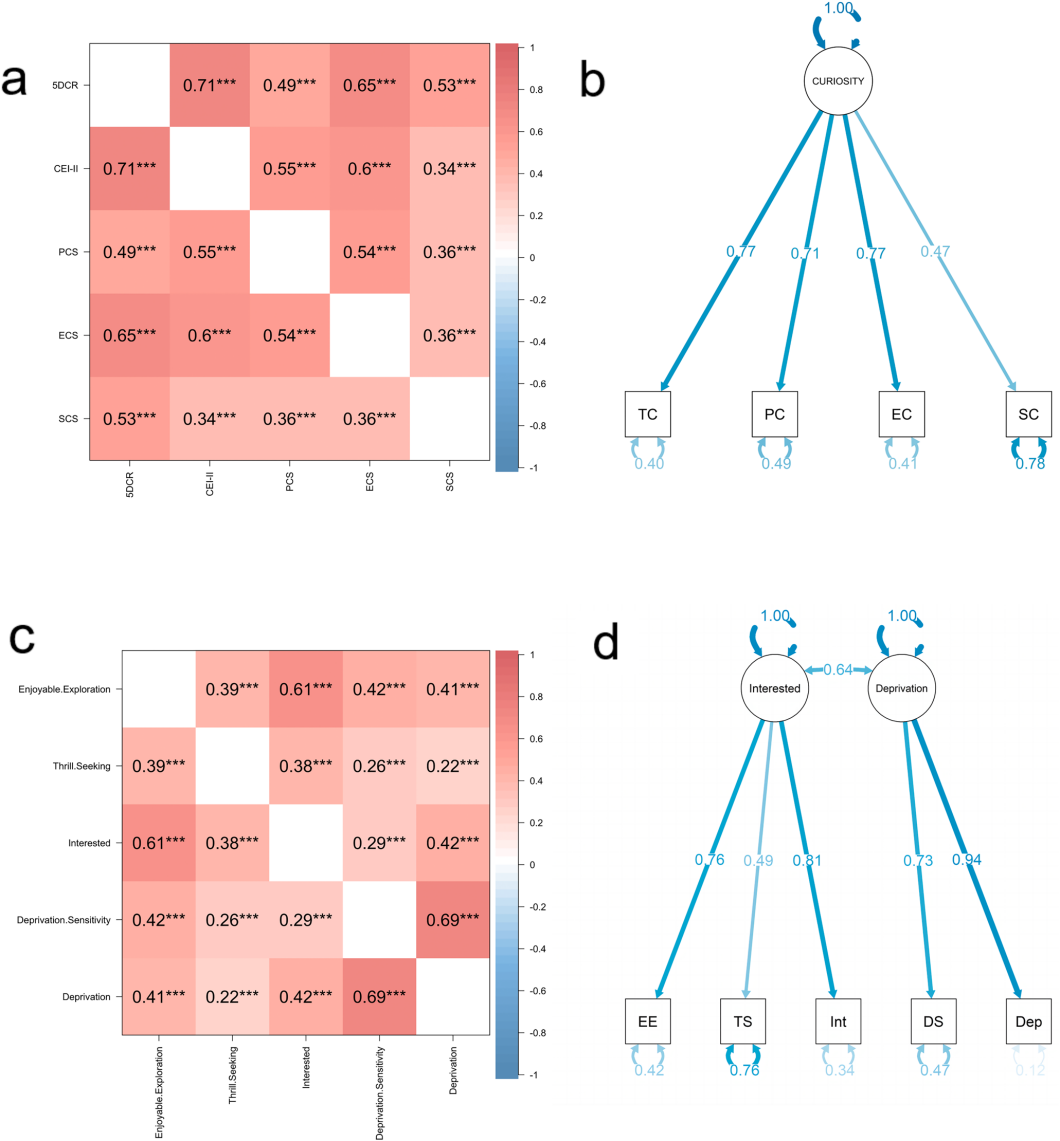
(a) The correlation matrix among different kinds of curiosity. 5DCR: Five‐Dimensional Curiosity Scale Revised; CEI‐II: Curiosity Exploration Inventory‐II; ECS: Epistemic Curiosity Scale; PCS: Perceptual Curiosity Scale; SCS: Social Curiosity Scale. (b) Standardized factor loadings and inter‐factor correlations for the one‐factor curiosity model for the current sample (*n* = 723). EC: epistemic curiosity; PC: perceptual curiosity; SC: social curiosity; TC: trait curiosity. (c) The correlation matrix among dimensions of Five‐Dimensional Curiosity Scale Revised and Epistemic Curiosity Scale. *Enjoyable Exploration*, *Thrill Thinking*, and *Deprivation Sensitivity* are dimensions of the Five‐Dimensional Curiosity Scale Revised; *Interested* and *Deprivation* are dimensions of the Epistemic Curiosity Scale. (d) Standardized factor loadings and inter‐factor correlations for the two‐factor interested‐deprivation curiosity model for the current sample (*n* = 723). Dep: deprivation; DS: deprivation sensitivity; EE: enjoyable exploration; Int: interested; TS: thrill seeking. ***p < 0.001.

Figure 4a shows highly positive correlations among perceptual curiosity, epistemic curiosity, social curiosity, trait curiosity, and five‐dimensional curiosity. These results may demonstrate that they measured similar concepts and reflect the different aspects of curiosity. We then fitted a model the same as Riedl et al. (2021) constructed, which contained CEI‐ II, PCS, ECS, and SCS to find a univariate latent concept from different kinds of curiosity. The model showed a good fit to the data according to the model fit indices (*χ* ^2^ (2, 723) = 2.43, *p* = 0.30; CFI = 1.00; TLI = 1.00; RMSEA = 0.02; SRMR = 0.01, NNFI = 1.00) (Figure 4b). The standardized factor loadings in this model range between 0.47 and 0.77 (all *p*s < 0.001), with an average variance extracted of 68%. We then analyzed the correlations among the scales' dimensions to test other curiosity taxonomies. From the definition of these dimensions of scales, the *Enjoyable Exploration* in 5DCR, *Stretching* in CEI‐II, *Diversive* in PCS, *Interested* in ECS, and *General* in SCS were similar, while *Deprivation Sensitivity* in 5DCR, *Embracing* in CEI‐II, *Specific* in PCS, *Deprivation* in ECS and *Covert* in SCS were similar (Figure 4c). These results suggested that curiosity can be classified not only according to the object of curiosity, but also according to whether it is specific or general, interested or deprived, and the latter two classifications may be highly similar. Then we constructed and fitted the interested‐deprivation curiosity model, which contains *Enjoyable Exploration, Deprivation Sensitivity, Thrill Seeking* dimensions in 5DCR, and *Interest* and *Deprivation* dimensions in ECS. All model fit indices except RMSEA indicated a good fit of this model (*χ* ^2^ (4, 723) = 40.84, *p* < 0.001; CFI = 0.97; TLI = 0.92; RMSEA = 0.11; SRMR = 0.02, NNFI = 0.92) (Figure 4d). The standardized factor loadings in this model ranged between 0.48 and 0.88 (all *p*s < 0.001), with an average variance extracted of 71%.

#### 3.2.2 Need for Cognition, Intolerance of Uncertainty, and Curiosity

**FIGURE 5:**
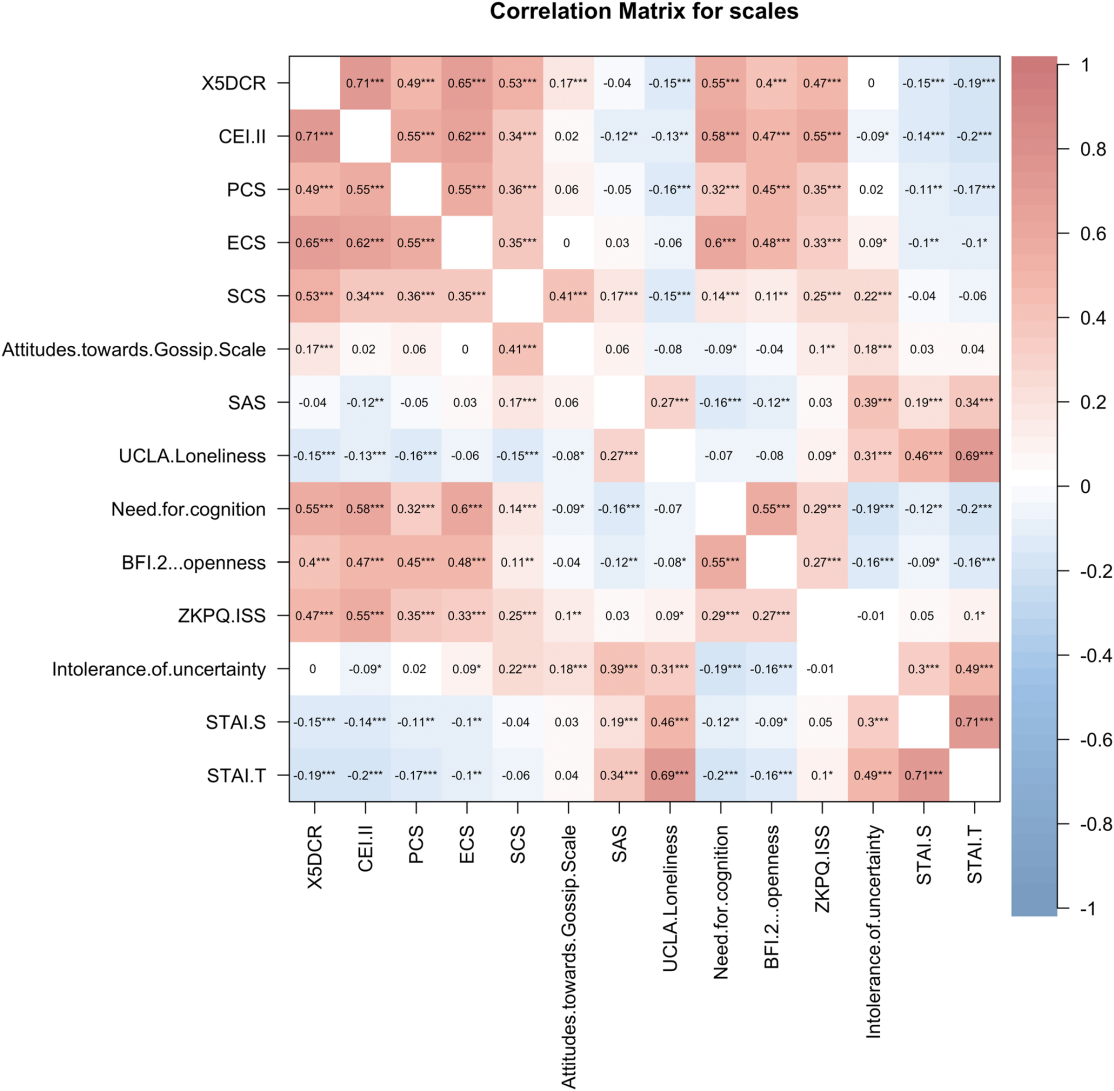
The correlation matrix of all scales in this study. BFI.2: Big Five Inventory‐2; CEI‐II: Curiosity Exploration Inventory‐II; ECS: Epistemic Curiosity Scale; PCS: Perceptual Curiosity Scale; SAS: Social Anxiety Scale; SCS: Social Curiosity Scale; STAI.S: State–Trait Anxiety Inventory‐State; STAI‐T: State–Trait Anxiety Inventory‐Trait; X5DCR(5DCR): Five‐Dimensional Curiosity Scale Revised; ZKPQ.ISS: Zuckerman–Kuhlman Personality Questionnaire, Impulsive Sensation Seeking. *p < 0.05; **p < 0.01; ***p < 0.001.

Figure 5 shows the correlation matrix of all scales in this study. The results showed that the need for cognition is highly correlated with 5DCR, trait curiosity and epistemic curiosity (5DCR: *r* = 0.55, *p* < 0.001; CEI‐II: *r* = 0.58, *p* < 0.001; ECS: *r* = 0.60, *p* < 0.001; Benjamin and Hochberg's *FDR* correction corrected), moderately correlated to perceptual curiosity, social curiosity (PCS: *r* = 0.32, *p* < 0.001; SCS: *r* = 0.14, *p* < 0.001; *FDR* corrected). Intolerance of uncertainty is only correlated with epistemic curiosity, social curiosity (ECS: *r* = 0.09, *p* = 0.01; SCS: *r* = 0.22, *p* < 0.001; *FDR* corrected), and not significantly correlated with 5DCR, perceptual curiosity (5DCR: *p* = 0.93; PCS: *p* = 0.63; *FDR* corrected), except trait curiosity (CEI‐II: *r* = −0.08, *p* = 0.02; *FDR* corrected). This result indicated that intolerance of uncertainty might be one of the drives to know information about others' behavior, emotions, and thoughts. Furthermore, both *Impulsive Sensation Seeking* in ZKPQ and *Openness* in BFI‐2 correlated with all kinds of curiosity, indicating impulsive sensation seeking and openness were two of the most important personality traits for individual curiosity. The multivariate regression analysis showed that impulsive sensation seeking, need for cognition, and openness trait could jointly predict trait curiosity (impulsive sensation seeking: *β* = 0.667, *p* < 0.001; need for cognition: *β* = 0.25, *p* < 0.001; openness trait: *β* = 0.13, *p* < 0.001, *R* ^2^ = 0.52, Figure 7a), these predictor variables did not exhibit significant multicollinearity (VIF = 1.12–1.47, all VIFs < 10).

#### 3.2.3 A Unique and Crucial Kind of Curiosity: Social Curiosity

**FIGURE 6:**
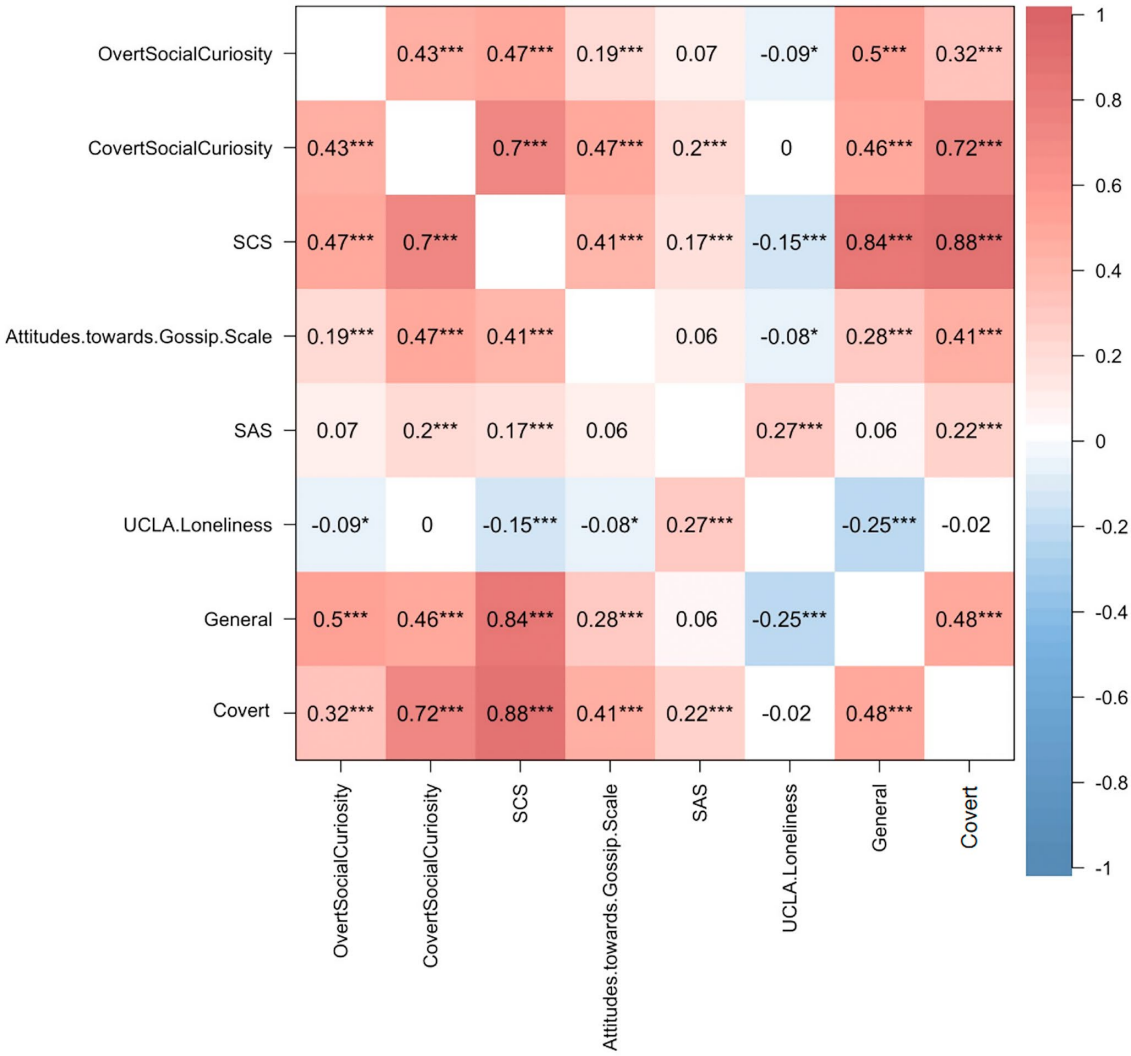
The correlation matrix between social curiosity and other scales. *Over Social Curiosity* and *Covert Social Curiosity* are dimensions of the Five‐Dimensional Curiosity Scale Revised. SAS: Social Anxiety Scale; SCS: Social Curiosity Scale. *General* and *Covert* are dimensions of the Social Curiosity Scale. *p < 0.05; **p < 0.01; ***p < 0.001.

Figure 6 shows the correlation between social curiosity and other scales. Our results indicated a negative correlation between social curiosity and loneliness (*r* = −0.15, *p* < 0.001; *FDR* corrected). The *General* dimension in SCS was correlated with loneliness (*r* = −0.25, *p* < 0.001), and the *Covert* dimension in SCS was correlated with social anxiety (*r* = 0.22, *p* < 0.001). This might imply that higher loneliness was negatively correlated with curiosity about other people, while higher social anxiety might correlate with internal motivations for surreptitiously observing and eavesdropping on others.

**FIGURE 7:**
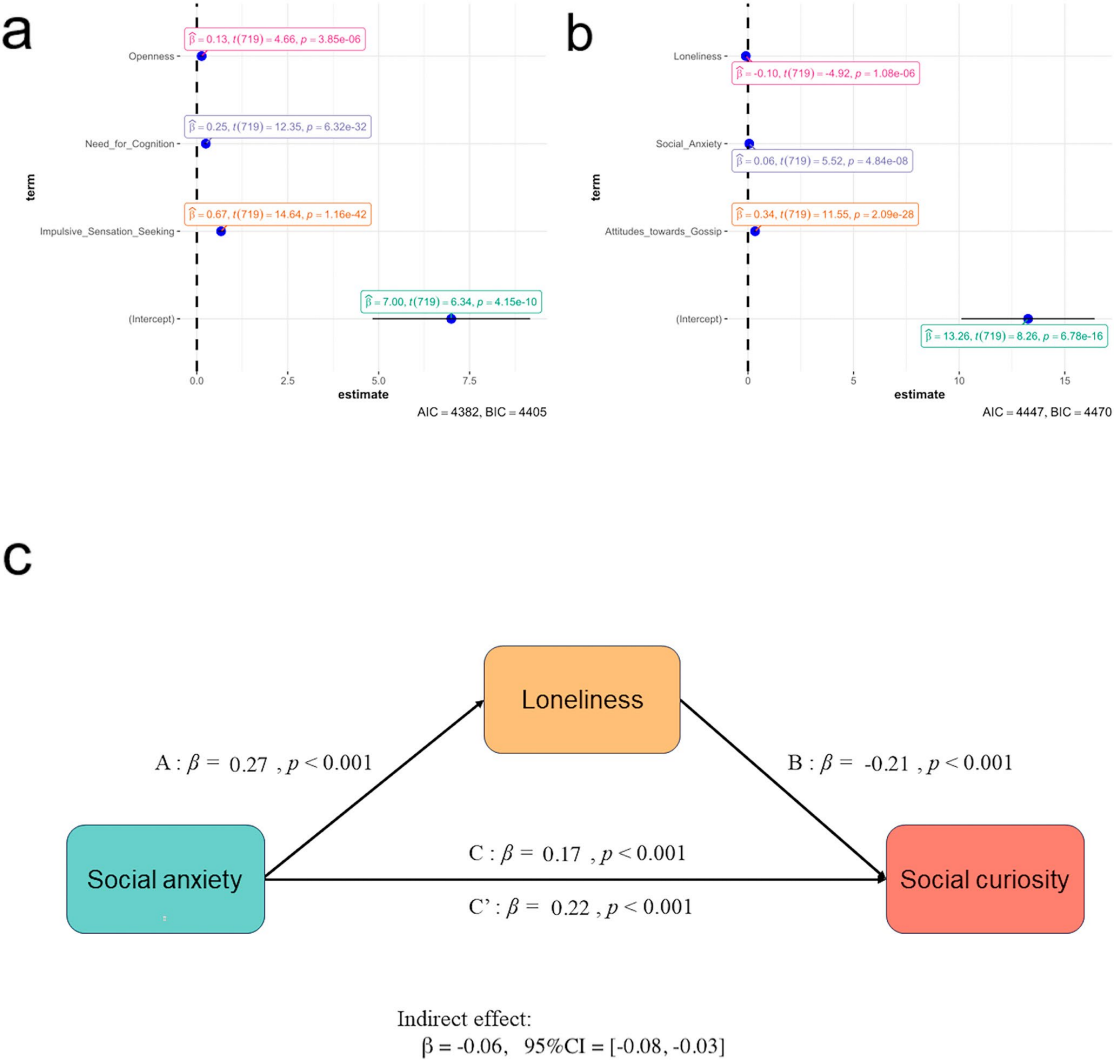
(a) The result of multivariate regression analysis among openness, need for cognition and impulsive sensation seeking. (b) The result of multivariate regression analysis among loneliness, social anxiety and attitudes toward gossip. (c) The mediation model. The relationship between social anxiety and social curiosity is partially mediated by loneliness. In the mediation model, loneliness partially mediates the relationship between social anxiety and social curiosity (indirect effect: *β* = −0.06, 95% CI: [−0.08, −0.03]).

The multivariate regression analysis showed that social anxiety, attitudes toward gossip, and loneliness can jointly predict social curiosity (social anxiety: *β* = 0.06, *p* < 0.001; attitudes toward gossip: *β* = 0.34, *p* < 0.001; loneliness: *β* = −0.1, *p* < 0.001, *R* ^2^ = 0.21) (Figure 7b). These predictor variables did not exhibit significant multicollinearity (VIF = 1.01–1.09, all VIFs < 10). Furthermore, Figure 7c shows the coefficient and 95% CI for each path, total effect, and direct effect in the mediation model. The relationship between social anxiety and social curiosity was partially mediated by loneliness (*β* = −0.06, 95% CI: [−0.08, −0.03], indirect effect).

#### 3.2.4 Emotions, Mood, and Curiosity

Figure 5 shows that 5DCR, trait curiosity, perceptual curiosity, and epistemic curiosity were correlated with state and trait anxiety. Besides, we found that loneliness was negatively correlated with 5DCR, trait curiosity, and perceptual curiosity (5DCR: *r* = −0.15, *p* < 0.001; CEI‐II: *r* = −0.13, *p* < 0.001; PCS: *r* = −0.16, *p* < 0.001; *FDR* corrected). These results suggest that emotional states like anxiety and loneliness would impede one's desire to know novel and unknown information.

## 4 General Discussion

### 4.1 The Validated Measures of Multifaceted Curiosity

The study presents the validity of translated Chinese curiosity scales, accesses their reliability, and compares the results with the original samples to verify the inspections. The calculated Cronbach's α and McDonald's ω coefficients, along with confirmatory factor analysis results, demonstrate the well‐established structural validity of Chinese scales, demonstrating their strong psychometric quality. The research also validates the previous taxonomy of curiosity and distinguishes between specificity and generalized curiosity. Additionally, it uncovers correlations among different types of curiosity, supporting the notion that different types of curiosity are both independent and partially related. Overall, the study provides effective curiosity tools for measuring curiosity and promotes the development of curiosity research in Chinese contexts.

### 4.2 Cognitive Components of Curiosity and Its Relationship With Other Scales

Current curiosity research is actively exploring the cognitive components of curiosity (Gruber and Ranganath 2019; Kidd and Hayden 2015; Kidd, Piantadosi, and Aslin 2012). Individuals have a need to understand and make the experiential world reasonable (Cacioppo and Petty 1982). However, when people have limited knowledge about their surroundings, they may feel uncertain and generate prediction errors between what they already know and the level of knowledge they aspire to achieve. All these feelings can further induce individual curiosity (Huang, Chen, et al. 2021; Kidd and Hayden 2015; Loewenstein 1994). Consistent with previous results (Li and Browne 2006; Olson, Camp, and Fuller 1984; Byman 2005; Marvin, Tedeschi, and Shohamy 2020), our analysis shows that both the need for cognition and impulsive sensation seeking are significant predictors of trait curiosity. Furthermore, our correlation results demonstrate that both the need for cognition and impulsive sensation seeking significantly correlate with perceptual and epistemic curiosity. This suggests that the cognitive components of curiosity refer to a deep desire to acquire knowledge and a tendency to be drawn to novel stimuli.

### 4.3 Curiosity in the Social Context

Social curiosity, defined as an interest in understanding human behavior, plays a crucial role in social interactions (Renner 2006). Similarly, the desire to engage in gossip, as a form of conversation about social topics, motivates adaptation to social environment (Foster 2004). Our analysis shows that attitudes toward gossip significantly predict social curiosity, consistent with previous results (Hartung and Renner 2013). As a fundamental human trait, social curiosity is considered unique due to its focus on exploring and understanding social interactions, relationships, and dynamics among individuals (Foster 2004; Kashdan and Roberts 2006; Sinha, Bai, and Cassell 2017). Therefore, understanding the interactions between social curiosity and other factors requires a comprehensive measurement of individual differences and contextual influences. The current study finds a mediating effect of loneliness on the relationship between social anxiety and social curiosity. It is suggested that individuals with high social anxiety may avoid social interaction due to fear of negative evaluation or judgment (Hofmann 2007; Leary and Jongman‐Sereno 2014). This avoidance can lead to social isolation and loneliness, which may reduce the likelihood of engaging in exploratory behaviors in social contexts, leading to the result of curiosity inhibition (Lim et al. 2016; Kashdan 2002; Jones, Rose, and Russell 1990). Given the potential differences in cognitive mechanisms between social curiosity and other forms of curiosity, future investigation into social curiosity should consider both social contexts and individual differences.

### 4.4 Emotions and Curiosity

The interplay between unfamiliar experiences and emotional responses, such as anxiety and curiosity, is intricate and influenced by factors like contradictions with existing knowledge, feelings of lack of personal control, and the natural tendency to seek potential rewards and personal growth. (Berlyne 1971; Kashdan and Fincham 2004; Molinaro and Collins 2023). Notably, these emotional responses can be further impacted by some curiosity elicitation and relief‐related emotions or mental states, such as fear, confidence, surprise, and disappointment, which can lead to an extended duration of emotional experiences (Zhu et al. 2019; Kaneko, Ozaki, and Horike 2018). From the perspective of the classification of reasons for curiosity, interest‐based curiosity is negatively correlated with anxiety, while deprivation‐based curiosity is positively correlated with anxiety (Litman 2010; Litman and Jimerson 2004). From the perspective of the stability of curiosity, trait and state curiosity are both negatively correlated with social anxiety (Kashdan and Roberts 2004, 2006). Besides, our results show that trait curiosity is significantly negatively correlated with both trait anxiety, state anxiety, and social anxiety, which is consistent with the previous results (Huang, Cao, et al. 2021). In the assessment of prediction error, if an anxiety state is induced, a psychological defense mechanism is developed that inhibits curiosity and exploratory behavior (Gruber and Ranganath 2019). Thus, our findings suggest that an emotional state of anxiety may hinder an individual's desire to know novel and unknown information.

Further, our results show that social anxiety is negatively associated with trait curiosity, consistent with previous research (Kashdan 2004). However, we also find that social anxiety is positively related to covert social curiosity. This is supported by the findings of Renner (2006), who suggests that social anxiety facilitates covert social curiosity. Additionally, recent research also finds individuals with a higher tendency to worry show greater willingness to explore (Witte et al. 2024). We provide further evidence of the relationship between social anxiety and social curiosity. However, the relationship between anxiety and curiosity appears to yield mixed results, depending on the specific taxonomy of curiosity considered (Smith et al. 2022; Aberg, Toren, and Paz 2022).

Some positive emotions play a crucial role in enhancing an individual's desire to explore the unknown and in facilitating curiosity (Deci and Ryan 1985; Fredrickson 2001). For example, Silvia (2008) specifically demonstrates “interest” as an emotion that directly enhances curiosity, leading to a greater engagement with novel stimuli and exploratory behavior. Our findings show that both openness traits and need for cognition are positively correlated with curiosity. This aligns with the previous research, which suggests that openness and need for cognition are associated with a preference for novelty and engaging in effortful activities, both of which are strongly linked to the feeling of interest (Feist 2012). Thus, interest is considered as a crucial emotional state that encourages continuous exploratory behavior and promotes curiosity.

### 4.5 Broader Issues and Future Directions

As for the limitations of the study, one concern arises about the participants' exposure to multiple curiosity scales online, which might lead to fatigue in providing ratings, even without overlapping items. Another consideration is whether samples from these online platforms yield high‐quality data due to potential issues with attention and effort. However, it is worth noting that online experiments provide a relaxed environment, flexibility in data collection, and the ability to capture naturalistic self‐report. To address potential issues, future research could consider offline experiments and avoiding excessively similar items for the same participant. In addition, the present study tries to explain the mechanisms of curiosity through the intercorrelation among different curiosity scales and its relationship with other psychological factors. Given the multidimensional nature of curiosity, which allows for studying from various perspectives, future studies could utilize these scales in conjunction with large‐scale testing and longitudinal development studies. As different categories of curiosity may predict curiosity‐relevant decisions differently, future research can explore the correlation between assessments and actual decisions or behaviors. Additionally, the investigation of how various curiosity categories can predict relevant decisions and behaviors, also highlights directions for future research, such as the exploration of the neural mechanisms connecting curiosity assessment and artificial intelligence.

## 5 Conclusion

This study translates several curiosity scales into Chinese and validates their reliability and structural validity against the original sample results. The comprehensive approach yields valuable insights into measuring different kinds of curiosity in the Chinese context, offering effective tools and potential interplay among curiosity and other psychological factors. The validation reveals consistently positive correlations among various curiosity scales. Additionally, we provide new evidence on the mechanisms of curiosity, suggesting that psychological factors such as the need for cognition and impulsive sensation seeking are underlying drives to curiosity.

## Conflicts of Interest

The authors declare no conflicts of interest.

## Supporting information


Data S1.

## Data Availability

The data that support the findings of this study are available at https://osf.io/efk2b/. The analysis code of the behavioral data is available at https://osf.io/efk2b/.
