# Supplementary material for: Unraveling the Intricacies of Curiosity: A Comprehensive Study of Its Measures in the Chinese Context
Source: Psych J. 2024 Nov 20;14(2):219–34. doi: 10.1002/pchj.813 (PMC11961245; doi:10.1002/pchj.813)
Supplement: Supplementary file 1 — Data S1. [file PCHJ-14-219-s001.docx]

**Scale Directory**

[1 五维度好奇心量表-修订版(Five-Dimensional Curiosity Scale Revised) 2](#_Toc154912657)

[2 好奇心与探索量表-II(The Curiosity and Exploration Inventory-II) 3](#_Toc154912658)

[3 知觉好奇心量表(Perceptual Curiosity Scale) 4](#_Toc154912659)

[4 认知好奇心量表(Epistemic Curiosity Scale) 5](#_Toc154912660)

[5 社会好奇心量表(Social Curiosity Scale) 6](#_Toc154912661)

[6 12项八卦态度量表(12-item Attitudes Towards Gossip) 7](#_Toc154912662)

[7 社会焦虑问卷(Social Anxiety Questionnaire) 8](#_Toc154912663)

[8 UCLA孤独感量表(The UCLA Loneliness Scale (Version 3)) 9](#_Toc154912664)

[9 认知需求量表(Need for Cognition Scale) 10](#_Toc154912665)

[10 大五人格量表-2 （开放性）(Big Five Inventory-2 (Openness)) 11](#_Toc154912666)

[11 冲动感觉寻求量表(Impulsive Sensation Seeking Scale) 12](#_Toc154912667)

[12 12项不确定性耐受度量表(The 12-item Intolerance of Uncertainty Scale) 13](#_Toc154912668)

[13 状态-特质焦虑量表的状态焦虑分量表（STAI-S)(State-Trait Anxiety Inventory: State anxiety subscale) 14](#_Toc154912669)

[14 状态-特质焦虑量表的特质焦虑分量表（STAI-T)(State-Trait Anxiety Inventory: Trait anxiety subscale) 15](#_Toc154912670)

[15 Five-Dimensional Curiosity Scale Revised (5DCR) 16](#_Toc154912671)

[16 The Curiosity and Exploration Inventory-II 17](#_Toc154912672)

[17 Perceptual Curiosity Scale 18](#_Toc154912673)

[18 Epistemic Curiosity Scale 19](#_Toc154912674)

[19 Social Curiosity Scale 20](#_Toc154912675)

[20 12-item Attitudes Towards Gossip 21](#_Toc154912676)

[21 Social Anxiety Questionnaire 22](#_Toc154912677)

[22 The UCLA Loneliness Scale (Version 3) 23](#_Toc154912678)

[23 Need for Cognition Scale 24](#_Toc154912679)

[24 Big Five Inventory-2 (Openness) 25](#_Toc154912680)

[25 Impulsive Sensation Seeking Scale 26](#_Toc154912681)

[26 The 12-item Intolerance of Uncertainty Scale 27](#_Toc154912682)

[27 State-Trait Anxiety Inventory: State anxiety subscale 28](#_Toc154912683)

[28 State-Trait Anxiety Inventory: Trait anxiety subscale 29](#_Toc154912684)

# 五维度好奇心量表-修订版 (Five-Dimensional Curiosity Scale Revised)

**指导语**：下面是人们经常用来描述自己的一些句子，请评估这些句子与你自身实际情况的符合程度。答案没有对错之分。

| 1 | 2 | 3 | 4 | 5 | 6 | 7 |
| --- | --- | --- | --- | --- | --- | --- |
| 完全不符合我 | 基本不符合我 | 有点不符合我 | 中等 | 一般符合我 | 基本符合我 | 完全符合我 |

1. 我会把具有挑战性的情景看作是一个成长和学习的机会
2. 我会寻求可能需要进入深度思考的状态
3. 我喜欢学习我不熟悉的科目
4. 我发现获取新信息很有趣
5. 思考概念性难题的解决方法会让我夜不能寐
6. 我可以花几个小时在一个问题上，因为不得到答案我没法休息
7. 如果我想不出问题的解决方法，我会感到沮丧，因此我会更加努力地去解决它
8. 我总是孜孜不倦地解决那些我认为必须解决的问题
9. 很小的问题都会阻止我寻求新的体验 （反向计分）
10. 我无法处理来自不确定情况的压力（反向计分）
11. 我发现当我对自己的能力缺乏信心时，我很难去探索新的领域（反向计分）
12. 当我有可能被吓到时，我很难集中注意力（反向计分）
13. 冒险令我感到兴奋
14. 当我有空的时候，我想做一些有点可怕的事情
15. 前行中不断创造冒险比有计划的冒险更有吸引力
16. 我更喜欢那些活跃但难以捉摸的朋友
17. 我通过问别人很多问题来知道别人对什么感兴趣
18. 当与兴奋的人交谈时，我会好奇地想找出他们兴奋的原因。
19. 当我和别人交谈时，我会努力去发现他们身上有趣的细节
20. 当人们做某件事时，我喜欢弄清楚他们为什么会这么做
21. 当别人在谈话时，我想要了解他们谈话的内容
22. 当周围有其他人在谈话时，我喜欢听他们的谈话
23. 当人们吵架的时候，我想要知道是怎么回事
24. 我会搜寻生活中的人们的私人生活信息

**维度说明**：

愉悦探索：1、2、3、4

信息匮乏敏感性：5、6、7、8

抗压能力：（整个分量表反向评分）：9、10、11、12

刺激寻求：13、14、15、16

社会好奇心：

公开性社会好奇心：17、18、19、20

隐蔽性社会好奇心：21、22、23、24

**计分说明**

7点评分。计算每个维度的平均项目得分，并分别进行分析（抗压能力项目反向计分）。

# 好奇心与探索量表-II (The Curiosity and Exploration Inventory-II)

**指导语**：根据你平时的感觉和行为方式，对一下叙述进行评分。注意，不要根据你认为应该怎么做，希望怎么做或不想怎么做来评价。请保证诚实作答。

| 1 | 2 | 3 | 4 | 5 |
| --- | --- | --- | --- | --- |
| 略有一点或根本没有 | 较少这样 | 中等 | 较多时候这样 | 经常或总是这样 |

1. 在新环境下，我会积极寻求尽可能多的信息
2. 我是那种很享受日常生活中的不确定性的人
3. 做一些复杂或具有挑战性的事情时，我处于最佳状态
4. 无论走到哪里，我都在寻找新事物或新体验
5. 我认为充满挑战的情境是成长和学习的机会
6. 我喜欢做一些有点令人害怕的事情
7. 我一直在寻找挑战我对自己和世界的看法的经历
8. 我更喜欢难以预测的工作
9. 我经常寻求挑战自己的机会，使自己成长
10. 我是那种乐于接受陌生的人，事物和地点的人

**维度说明**：

延伸（寻求知识和新体验的动机）：1，3，5，7，9

拥抱（愿意接受日常生活中新颖、不确定和不可预知）：2，4，6，8，10

**计分说明**：5点评分，全部正向计分。

# 知觉好奇心量表 (Perceptual Curiosity Scale)

**指导语**：下面有一些描述，请你阅读这些描述，并且判断你平时在生活实际中做这些事情的频率。

| 1 | 2 | 3 | 4 |
| --- | --- | --- | --- |
| 几乎从不 | 有时 | 较多时候 | 几乎总是 |

1. 发现新的去处
2. 去从未去过的地方旅行
3. 听一些新类型或不常见类型的音乐
4. 探索我的周围环境
5. 尝试不同的食物
6. 去参观美术馆或博物馆
7. 闻到一些新的气味并弄清楚是什么
8. 听到一些奇怪的声音并找出引起它的原因
9. 看到新事物并触摸和感受它
10. 听到一些声音/动静并去看它是什么
11. 听到乐器的声音并乐意去看看它
12. 看到合唱团并去分辨其中不同音色类型

**删除项**：

1. 观看艺术展览
2. 穿过有趣的建筑
3. 想要去探索洞穴
4. 更愿意去从未去过的公园

**维度说明**：

发散性：1、2、3、4、5、6

具体化：7、8、9、10、11、12

项目 13-16 未注明分配给 PC/D 或 PC/S 分量表

**计分说明**：1-4李克特评分, 全部正向计分。

# 认知好奇心量表 (Epistemic Curiosity Scale)

**指导语**：下面有十条描述，请你阅读这些描述，并且判断你平时在生活实际中做这些事情的频率。

| 1 | 2 | 3 | 4 |
| --- | --- | --- | --- |
| 几乎从不 | 有时 | 较多时候 | 几乎总是 |

1.我喜欢探索新的观点和看法

2.我认为了解新的信息是一件很棒的事情

3.我喜欢去了解一些自己不太熟悉的事物

5.当认识到新事物时，我会想要去寻找更多与其相关的信息

6.我会花好几个小时在一个问题上，得不到答案我是不会休息的

7.我为了解决问题会苦思冥想

9.如果我不能解决问题，我会感到很沮丧，因此我会更加努力

10.我拼命解决那些我认为必须解决的问题

**删除项**：

4. 我喜欢讨论抽象的概念

8. 概念性的问题让我保持清醒地思考

**维度说明**：

兴趣型（多样性）：1、2、3、4、5

剥夺型（剥夺感的持续性）：6、7、8、9、10

**计分说明**：全部正向计分

# 社会好奇心量表 (Social Curiosity Scale)

**指导语**：下面有十条描述，请你根据对自己的总体看法，选择最符合的选项。

| 1 | 2 | 3 | 4 |
| --- | --- | --- | --- |
| 非常不同意 | 有点同意 | 同意 | 非常同意 |

1. 每当新认识一个人，我都会想更多地去了解有关这个人的信息
2. 我对人很感兴趣
3. 我认为认识新朋友是一件很棒的事情
4. 我喜欢去了解别人的习惯
5. 我喜欢去了解别人是如何工作的
6. 当别人在交谈时，我会想知道他们在聊什么
7. 在火车或者高铁上的时候，我喜欢听别人聊天
8. 我常常喜欢站在窗户边看邻居们正在做什么
9. 我喜欢往别人家亮着的窗户里看
10. 当人们争吵时，我想要知道发生了什么

**维度说明**：

一般好奇心：1、2、3、4、5

隐性好奇心：6、7、8、9、10

**计分说明**：全部正向计分，可以分维度计分，也可以记总分。

# 12项八卦态度量表 (12-item Attitudes Towards Gossip)

**指导语**：这些陈述旨在评估每个人对八卦的态度。你需要从以下五个选项中选择出你对每一条陈述的态度，分别是：非常同意，有些同意，中立，有些不同意，非常不同意。

| 1 | 2 | 3 | 4 | 5 |
| --- | --- | --- | --- | --- |
| 非常不同意 | 有点同意 | 中立 | 同意 | 非常同意 |

1. 我更关注工作而不是八卦 （反向计分）
2. 我想要知道别人的生活中发生了什么
3. 我喜欢跟别人分享自己听到的东西
4. 谈论别人很有趣
5. 聊八卦是一个消磨时间的好方法
6. 八卦是一个打破僵局的好方法
7. 不能相信八卦 （反向计分）
8. 从不知道八卦是有用的 （反向计分）
9. 八卦通常都是对的
10. 即使流言是真的也不应该被提起 （反向计分）
11. 流言很少是真的 （反向计分）
12. 谈论别人是不对的 （反向计分）

**维度说明**：

社会价值：1、2、3、4、5、6

道德价值：7、8、9、10、11、12

# 社会焦虑问卷 (Social Anxiety Questionnaire)

**指导语**：下面是一系列可能会或者不会让你感到不安、压力或紧张的社交场合。请就每一种社交场合选择符合你自身反应的选项。如果你从未经历过这些场合，请想象一下身处其中，你的不安、压力或者紧张程度会是什么水平，并选择相应选项。

| 1 | 2 | 3 | 4 | 5 |
| --- | --- | --- | --- | --- |
| 完全没有或非常轻微 | 轻微 | 中等 | 比较强烈 | 极度强烈 |

1. 跟别人打招呼却被忽视
2. 不得不去要求邻居停止制造噪音
3. 在公共场合讲话
4. 邀请有魅力的异性约会
5. 向服务员抱怨我的食物
6. 感觉自己被异性注视
7. 参加有权威人士在场的会议
8. 与人交谈时，那人不注意听我在讲什么
9. 当别人让我做我不喜欢做的事情时，我拒绝了他/她
10. 结交新朋友
11. 告诉别人他们令我伤心了
12. 不得不在班级里、工作中或者会议上讲话
13. 与一个刚刚认识的人保持交谈
14. 对一个挑我毛病的人表达我的恼怒
15. 在社交会上问候每一个人，而大部分的人我都不认识
16. 在公共场合被嘲笑
17. 在派对或者聚会上与我不认识的人说话
18. 上课时被老师提问或者在会议中被上级提问
19. 和一个刚认识的人聊天的时候，看着他/她的眼睛
20. 被我喜欢的人约出去
21. 在别人面前犯错
22. 参加一个社交活动，里面我只认识一个人
23. 主动和我喜欢的异性交谈
24. 我因为做错了事情受到斥责
25. 当和同学、同事或者工作伙伴一起吃饭时，被要求作为整组的代表发言
26. 告诉别人他们的行为打扰到了自己并请他们停下
27. 邀请我喜欢的人跳舞
28. 被批评
29. 与上级或者权威人士说话
30. 告诉我喜欢的人我想要更好地了解他们

# UCLA孤独感量表 (The UCLA Loneliness Scale (Version 3))

**指导语**：下面的陈述描述了人们有些时候的感觉。对于每句话，请根据你有多经常感受到句子所描述的感觉，选择最合适您实际情况的选项。下面是一个例子：

你多久感到一次快乐？

如果你从未感到快乐，就回答“从不”;如果你总是感到快乐，就回答“总是”

| 1 | 2 | 3 | 4 |
| --- | --- | --- | --- |
| 从不 | 很少 | 有时 | 总是 |

1. 你是否觉得自己与周围的人很合拍? (反向计分)
2. 你是否感到缺少陪伴?
3. 你是否觉得没有人可以求助?
4. 你是否感到孤独?
5. 你是否觉得自己是一群朋友中的一员? (反向计分)
6. 你是否觉得自己和周围的人有很多共同点? (反向计分)
7. 你是否觉得自己和别人不再亲近了?
8. 你是否觉得你的兴趣和想法与周围的人不同?
9. 你是否觉得自己外向友好? (反向计分)
10. 你是否觉得自己和别人很亲近? (反向计分)
11. 你是否感到自己被冷落?
12. 你是否觉得你与他人的关系毫无意义?
13. 你是否觉得没有人真正了解你?
14. 你是否感到被他人孤立?
15. 当你需要的时候，你是否能找到人陪伴? (反向计分)
16. 你是否觉得有人真正理解你? (反向计分)
17. 你是否感到害羞?
18. 你是否觉得别人在你身边却不能真正陪伴你?
19. 你是否觉得有人可以倾诉？(反向计分)
20. 你是否觉得有人可以求助？(反向计分)

# 认知需求量表 (Need for Cognition Scale)

**指导语**：对于下面的陈述，请指出这些陈述是否是你的特点或者是你所相信的。比如，如果这条陈述非常不符合你的特点或者与你对自己的看法完全不一样（一点也不符合你），请选择“非常不符合我”。如果这条陈述非常符合你的特点或者与你对自己的看法一样（非常符合你），请选择“非常符合我”。请使用下面的量表来评价以下每一条陈述。

| 1 | 2 | 3 | 4 | 5 |
| --- | --- | --- | --- | --- |
| 非常不符合我 | 不太符合我 | 不确定 | 有一点符合我 | 非常符合我 |

1. 我更喜欢复杂的问题而不是简单的问题
2. 我喜欢负责处理需要大量思考的情况
3. 思考并不是我的乐趣所在 (反向计分)
4. 我宁愿做不太需要思考的事情，也不愿做那些肯定会挑战我思考能力的事情 (反向计分)
5. 我试图预测并避免那些我不得不进行深入思考的事情 (反向计分)
6. 我能在长时间的努力思考中找到满足感
7. 我只在必要的时候努力思考 (反向计分)
8. 我更喜欢思考日常的小项目而不是长期项目 (反向计分)
9. 我喜欢那些我一旦学会就不太需要思考的任务 (反向计分)
10. 依靠思考登上人生巅峰这种想法对我很有吸引力
11. 我非常喜欢能够对某个问题贡献新解决方法的任务
12. 学习新的思考方式并不能使我感到兴奋 (反向计分)
13. 我喜欢我的生活充满了我必须解决的难题
14. 抽象思维的概念很吸引我
15. 我宁愿选择凭理智做事、难度大、重要的任务，而不愿选择有一些重要但不需要太多思考的任务
16. 在完成一项需要大量脑力劳动的任务后，我感到的是解脱而不是满足 (反向计分)
17. 对我来说，只要有什么东西能完成任务就足够了；至于它是怎么运作的或为何这样运作，我并不在乎 (反向计分)
18. 我经常会仔细考虑一些问题，即使这些问题并不影响我

# 大五人格量表-2 （开放性）(Big Five Inventory-2 (Openness))

**指导语**：下面是一些关于个人特征的描述，有些可能适用于你，有些可能不适用于你。请选择对应的选项以表明你同意或不同意这个描述。

| 1 | 2 | 3 | 4 | 5 |
| --- | --- | --- | --- | --- |
| 非常不同意 | 不太同意 | 态度中立 | 比较同意 | 非常同意 |

1. 对艺术没什么兴趣 （反向计分）
2. 对许多不同的事物都感兴趣
3. 善于创造，能找到聪明的方法来做事
4. 着迷于艺术、音乐或文学
5. 不喜欢知识性或者哲学性强的讨论 （反向计分）
6. 几乎没有什么创造性 （反向计分）
7. 重视艺术与审美
8. 思想深刻
9. 缺乏想象力（反向计分）
10. 觉得诗歌、戏剧很无聊（反向计分）
11. 对抽象的概念和想法没什么兴趣（反向计分）
12. 有创意，能想出新点子

# 冲动感觉寻求量表 (Impulsive Sensation Seeking Scale)

**指导语**：对于以下每句话，请选择最经常感受所对应的选项。

| 0 | 1 |
| --- | --- |
| 否 | 是 |

1. 我喜欢有新的、令人兴奋的经历和感觉，即使它们有点可怕
2. 我喜欢为了刺激而做事
3. 我有时会做一些“疯狂“的事情，只是为了好玩
4. 我有时喜欢做一些有点吓人的事情
5. 我喜欢进入那种无法预测事情的结果的新的环境
6. 我喜欢什么都尝试一次
7. 我更喜欢那些难以捉摸又令人兴奋的朋友
8. 我喜欢“狂野“不羁的派对
9. 我喜欢那种到处奔波，到处旅行，充满变化和兴奋的生活
10. 我是个容易冲动的人
11. 我喜欢独自探索一个陌生的城市或城镇的一部分，即使有可能会迷路
12. 我想在没有预先计划或确定的路线或时间表的情况下出发旅行
13. 在我开始一项复杂的工作之前，我会做仔细的计划 （反向计分）
14. 我很少花很多时间在提前计划的细节上
15. 我倾向于在开始一项新工作之前，不制定太多计划
16. 我通常在做事情之前会考虑我要做什么（反向计分）
17. 我经常冲动行事
18. 我经常被新鲜的、令人兴奋的东西和想法冲昏头脑，从不考虑可能发生的复杂情况
19. 我倾向于经常改变我的兴趣

# 12项不确定性耐受度量表 (The 12-item Intolerance of Uncertainty Scale)

**指导语**：请评价您对以下叙述的同意程度，并选择最合适的答案。

| 1 | 2 | 3 | 4 | 5 |
| --- | --- | --- | --- | --- |
| 非常不符合我 | 有一点符合我 | 比较符合我 | 非常符合我 | 完全符合我 |

1. 无法预见的事件让我非常不安
2. 没有掌握我需要的所有信息会让我很沮丧
3. 一个人应始终多想一步，以避免意外
4. 即使有最好的计划，一个小的、无法预见的事件也可能会破坏一切
5. 我一直想知道未来会发生什么
6. 我无法忍受出其不意的事情
7. 我必须有能力提前安排好一切才行
8. 不确定性使我无法过上充实的生活
9. 当需要采取行动时，不确定性使我丧失行动力
10. 当我面临不确定时，我不能很好地做出行动
11. 一点点的疑惑都能妨碍我做出行动
12. 我必须摆脱所有不确定的情况

# 状态-特质焦虑量表的状态焦虑分量表（STAI-S) (State-Trait Anxiety Inventory: State anxiety subscale)

**指导语**：下面列出的是一些人们常常用来描述自己的句子，请阅读每一个句子，然后选择最符合你此时此刻的感觉的选项。不存在正确或错误的选项，因此请不要花太多的时间去考虑，选择最符合你当下的感觉的选项即可

| 1 | 2 | 3 | 4 |
| --- | --- | --- | --- |
| 完全没有 | 有些 | 中等程度 | 非常明显 |

1.我感到心情平静 （反向计分）

2.我感到安全（反向计分）

3.我是紧张的

4.我感到有束缚感

5.我感到安逸（反向计分）

6.我感到心烦意乱

7.我正在为可能发生的不幸而忧虑

8.我感到满意（反向计分）

9.我感到害怕

10.我感到舒适（反向计分）

11.我感到自信（反向计分）

12.我感到紧张

13.我非常紧张不安

14.我优柔寡断

15.我感到轻松（反向计分）

16.我感到心满意足（反向计分）

17.我感到担忧

18.我感到困惑

19.我感到镇定（反向计分）

20.我感到愉快（反向计分）

# 状态-特质焦虑量表的特质焦虑分量表（STAI-T) (State-Trait Anxiety Inventory: Trait anxiety subscale)

**指导语**：以下是人们用来描述自己的一些句子。请阅读每个句子，并根据自己平时的一般感受选择符合自己情况的选项。

| 1 | 2 | 3 | 4 |
| --- | --- | --- | --- |
| 几乎从不 | 有时 | 通常 | 几乎总是 |

1.我感到愉快（反向计分）

2.我感到神经和不安

3.我对自己感到满意（反向计分）

4.我希望我可以像其他人一样快乐

5.我觉得自己是个失败者

6.我感觉精力充沛（反向计分）

7.我“平静、冷静、沉着” （反向计分）

8.我觉得困难堆积如山，并且我无法克服他们

9.我为一些实际上无关紧要的事情担心太多

10.我感到高兴（反向计分）

11.我有令人不安的想法

12.我缺乏自信

13.我感到安全（反向计分）

14.我很容易做出决定（反向计分）

15.我觉得力不从心

16.我感到满足（反向计分）

17.一些不重要的想法在我的脑海中回荡，并困扰着我

18.我对我令别人失望的事很敏感，以至于我无法把它们从我的脑海中抹去

19.我是一个踏实稳重的人（反向计分）

20.当我思考我最近关注的问题和感兴趣的事物时，我就会陷入紧张不安的状态

# Five-Dimensional Curiosity Scale Revised (5DCR)

**Instructions**: Below are statements people often use to describe themselves. Please use the scale below to indicate the degree to which these statements accurately describe you. There are no right or wrong answers.

1 – Does not describe me at all

2 – Barely describes me

3 – Somewhat describes me

4 – Neutral

5 – Generally describes me

6 – Mostly describes me

7 – Completely describes me

1. I view challenging situations as an opportunity to grow and learn.
2. I seek out situations where it is likely that I will have to think in depth about something.
3. I enjoy learning about subjects that are unfamiliar to me.
4. I find it fascinating to learn new information.
5. Thinking about solutions to difficult conceptual problems can keep me awake at night.
6. I can spend hours on a single problem because I just can't rest without knowing the answer.
7. I feel frustrated if I can't figure out the solution to a problem, so I work even harder to solve it.
8. I work relentlessly at problems that I feel must be solved.
9. The smallest doubt can stop me from seeking out new experiences. (R)
10. I cannot handle the stress that comes from entering uncertain situations. (R)
11. I find it hard to explore new places when I lack confidence in my abilities. (R)
12. It is difficult to concentrate when there is a possibility that I will be taken by surprise. (R)
13. Risk-taking is exciting to me.
14. When I have free time, I want to do things that are a little scary.
15. Creating an adventure as I go is much more appealing than a planned adventure.
16. I prefer friends who are excitingly unpredictable.
17. I ask a lot of questions to figure out what interests other people.
18. When talking to someone who is excited, I am curious to find out why.
19. When talking to someone, I try to discover interesting details about them.
20. I like finding out why people behave the way they do.
21. When other people are having a conversation, I like to find out what it's about.
22. When around other people, I like listening to their conversations.
23. When people quarrel, I like to know what's going on.
24. I seek out information about the private lives of people in my life.

# The Curiosity and Exploration Inventory-II

**Instructions**: Rate the statements below for how accurately they reflect the way you generally feel and behave. Do not rate what you think you should do, or wish you do, or things you no longer do. Please be as honest as possible.

1. very slightly or not at all
2. a little
3. moderately
4. quite a bit
5. extremely
6. I actively seek as much information as I can in new situations.
7. I am the type of person who really enjoys the uncertainty of everyday life.
8. I am at my best when doing something that is complex or challenging.
9. Everywhere I go, I am out looking for new things or experiences.
10. I view challenging situations as an opportunity to grow and learn.
11. I like to do things that are a little frightening.
12. I am always looking for experiences that challenge how I think about myself and the world.
13. I prefer jobs that are excitingly unpredictable.
14. I frequently seek out opportunities to challenge myself and grow as a person.
15. I am the kind of person who embraces unfamiliar people, events, and places.

**Dimensions**:

Stretching (motivation to seek out knowledge and new experiences): 1, 3, 5, 7, 9

Embracing (willingness to embrace the novel, uncertain, and unpredictable nature of everyday life): 2, 4, 6, 8, 10

**Scoring method**: 5-point scale. No reversed scoring item.

# Perceptual Curiosity Scale

**Instructions**: There are some descriptions below. Please read these descriptions and judge how often you do these things in daily life. The rating alternatives were:

1. Almost Never
2. Sometimes
3. Often
4. Almost Always
5. Discover new places to go
6. Travel to places/never been to
7. Listen to new/unusual kinds of music
8. Exploring my surroundings
9. Enjoy trying different foods
10. Visiting art galleries/museums
11. Smell something new/find out what
12. Hear strange sound/find out what caused it
13. See new fabric/touch and feel it
14. Hear something/see what it is
15. Hear musical instrument/like to see it
16. See vocal group/different voice types

**Deleted items**:

1. Viewing art display
2. Walking through interesting buildings
3. Cave/want to explore
4. Rather visit a park/never been to

**Dimensions**:

Diversive: 1, 2, 3, 4, 5, 6

Specific: 7, 8, 9, 10, 11, 12

Item 13-16 could not be assigned meaningfully to either the PC/D or PC/S subscale

**Scoring method**: 1-4 Likert score All positive scores.

# Epistemic Curiosity Scale

**Instructions**: Participants were instructed to report how they ‘‘generally feel” regarding each item statement by rating themselves on the following 4- point frequency scale:

1. Almost Never
2. Sometimes
3. Often
4. Almost Always

1.Enjoy exploring new ideas

2.Find it fascinating to learn new information

3.Enjoy learning about subjects that are unfamiliar to me

5.Learn something new, like to find out more about it

6.Hours on a problem because I cannot rest without answer

7.Brood for a long time to solve problem

9.Frustrated if I cannot figure out problem, so I work even harder

10.Work like a fiend at problems that I feel must be solved

**Deleted items**:

4. Enjoy discussing abstract concepts

8. Conceptual problems keep me awake thinking about solutions

**Dimensions**:

I-type (EC-Diversive subscale items): 1, 2, 3, 4, 5

D-type (CFD-P subscale items): 6, 7, 8, 9, 10

**Scoring method**: All points are scored positively.

# Social Curiosity Scale

**Instructions**: Please report how you “generally perceive yourself” on a 4-point scale

1. Strongly disagree
2. Disagree
3. Agree
4. Strongly agree
5. When I meet a new person, I am interested in learning more about him/her
6. I’m interested in people
7. I find it fascinating to get to know new people
8. I like to learn about the habits of others
9. I like finding out how others “work”
10. When other people are having conversations, I like to find out what it’s about
11. When on the train, I like listening to other people’s conversations
12. Every so often I like to stand at the window and watch what my neighbors are doing
13. I like to look into other people’s lit windows
14. When people quarrel, I like to know what’s going on

**Dimensions**:

General social curiosity: 1, 2, 3, 4, 5

Covert social curiosity: 6, 7, 8, 9, 10

**Scoring method**: All positive scoring, scoring can be divided into dimensions, or total score can be recorded.

# 12-item Attitudes Towards Gossip

1. Strongly disagree
2. Disagree
3. Neutral
4. Agree
5. Strongly agree
6. Mind business instead of gossiping (R)
7. Love to know what is going on in people’s lives
8. Like to share what I hear
9. Fun to talk about people
10. Gossiping is a great way to pass time
11. Gossip is good ice-breaker
12. Cannot trust gossip (R)
13. Never known gossip to be helpful (R)
14. Gossip is often true
15. Never mention rumors even if true (R)
16. Rumors are hardly ever true (R)
17. Wrong to talk about others (R)

**Dimensions**:

Social Value: 1, 2, 3, 4, 5, 6

Moral Value: 7, 8, 9, 10, 11, 12

# Social Anxiety Questionnaire

**Instructions**: Below are a series of social situations that may or may not cause you unease, stress or nervousness. Please place an “X” on the number next to each social situation that best reflects your reaction, where “1” represents no unease, stress or nervousness and “5” represents very high or extreme unease stress, or nervousness.

If you have never experienced the situation described, please imagine what your level of unease, stress, or nervousness might be if you were in that situation and rate how you imagine you would feel by placing an “X” on the corresponding number

1. Not at all or very slight
2. Slight
3. Moderate
4. High
5. Very high or extremely high

1. Greeting someone and being ignored

2. Having to ask a neighbor to stop making noise

3. Speaking in public

4. Asking someone attractive of the opposite sex for a date

5. Complaining to the waiter about my food

6. Feeling watched by people of the opposite sex

7. Participating in a meeting with people in authority

8. Talking to someone who isn’t paying attention to what I am saying

9. Refusing when asked to do something I don’t like doing

10. Making new friends

11. Telling someone that they have hurt my feelings

12. Having to speak in class, at work, or in a meeting

13. Maintaining a conversation with someone I’ve just met

14. Expressing my annoyance to someone that is picking on me

15. Greeting each person at a social meeting when I don’t know most of them

16. Being teased in public

17. Talking to people I don’t know at a party or a meeting

18. Being asked a question in class by the teacher or by a superior in a meeting

19. Looking into the eyes of someone I have just met while we are talking

20. Being asked out by a person I am attracted to

21. Making a mistake in front of other people

22. Attending a social event where I know only one person

23. Starting a conversation with someone of the opposite sex that I like

24. Being reprimanded about something I have done wrong

25. While having dinner with colleagues, classmates or workmates, being asked

to speak on behalf of the entire group

26. Telling someone that their behavior bothers me and asking them to stop

27. Asking someone I find attractive to dance

28. Being criticized

29. Talking to a superior or a person in authority

30. Telling someone I am attracted to that I would like to get to know them better

# The UCLA Loneliness Scale (Version 3)

**Instructions**: The following statements describe how people are sometimes fed. For each statement, please indicate how often you feel the way described by writing a number in the space provided. Here is an example:

How often do you feel happy?

If you never felt happy, you would respond "never": if you always feel happy, you would respond "always."

1. Never
2. Rarely
3. Sometimes
4. Always
5. How often do you feel that you are “in tune” with the people around you? (R)
6. How often do you feel that you lack companionship?
7. How often do you feel that there is no one you can turn to?
8. How often do you feel alone?
9. How often do you feel part of a group of friends? (R)
10. How often do you feel that you have a lot in common with the people around you? (R)
11. How often do you feel that you are no longer close to anyone?
12. How often do you feel that your interests and ideas are not shared by those around you?
13. How often do you feel outgoing and friendly? (R)
14. How often do you feel close to people? (R)
15. How often do you feel left out?
16. How often do you feel that your relationships with others are not meaningful?
17. How often do you feel that no one really knows you well?
18. How often do you feel isolated from others?
19. How often do you feel that you can find companionship when you want it? (R)
20. How often do you feel that there are people who really understand you? (R)
21. How often do you feel shy?
22. How often do you feel that people are around you but not with you?
23. How often do you feel that there are people you can talk to? (R)
24. How often do you feel that there are people you can turn to? (R)

# Need for Cognition Scale

+4 = very strong agreement

+3 = strong agreement

+2 = moderate agreement

+1 = slight agreement

0 = neither agreement nor disagreement

-1 = slight disagreement

-2 = moderate disagreement

-3 = strong disagreement

-4 = very strong disagreement

1. I would prefer complex to simple problems.
2. I like to have the responsibility of handling a situation that requires a lot of thinking.
3. Thinking is not my idea of fun. (R)
4. I would rather do something that requires little thought than something that is sure to challenge my thinking abilities. (R)
5. I try to anticipate and avoid situations where there is likely a chance I will have to think in depth about something. (R)
6. I find satisfaction in deliberating hard and for long hours.
7. I only think as hard as I have to. (R)
8. I prefer to think about small, daily projects to long-term ones. (R)
9. I like tasks that require little thought once I’ve learned them. (R)
10. The idea of relying on thought to make my way to the top appeals to me.
11. I really enjoy a task that involves coming up with new solutions to problems.
12. Learning new ways to think doesn’t excite me very much. (R)
13. I prefer my life to be filled with puzzles that I must solve.
14. The notion of thinking abstractly is appealing to me.
15. I would prefer a task that is intellectual, difficult, and important to one that is somewhat important but does not require much thought.
16. I feel relief rather than satisfaction after completing a task that required a lot of mental effort. (R)
17. It’s enough for me that something gets the job done; I don’t care how or why it works. (R)
18. I usually end up deliberating about issues even when they do not affect me personally.

# Big Five Inventory-2 (Openness)

1. Disagree strongly
2. Disagree a little
3. Neutral
4. Agree a little
5. Agree strongly
6. Have few artistic interests (R)
7. Is curious about many different things
8. Is inventive, finds clever ways to do things
9. Is fascinated by art, music, or literature
10. Avoids intellectual, philosophical discussions (R)
11. Has little creativity (R)
12. Values art and beauty
13. Is complex, a deep thinker
14. Has difficulty imagining things (R)
15. Thinks poverty and plays are boring (R)
16. Has little interest in abstract ideas (R)
17. Is original, comes up with new ideas

# Impulsive Sensation Seeking Scale

1. No
2. Yes
3. I like to have new and exciting experiences and sensations, even if they might be a little scary to me
4. I like to do certain things just for the thrill of it
5. I sometimes do crazy things just for fun
6. I sometimes like to do things that are a little frightening
7. I enjoy getting into new situations where I can't predict how things will turn out
8. I'll try anything once
9. I prefer friends who are excitingly unpredictable
10. I like "wild" uninhibited parties
11. I would like the kind of life where one is on the move and traveling a lot, with lots of change and excitement
12. I am generally an impulsive person
13. I like to explore a strange city or section of town by myself, even if it means getting lost
14. I would like to take off on a trip with no preplanned or definite routes or timetable
15. Before I begin a complicated job or project, I tend to make careful plans (R)
16. I very seldom spend much time on the details of planning ahead
17. I tend to start a new task or project without much advance planning on how I will do it
18. I usually think about what I am going to do before I do it (R)
19. I tend to do things on impulse
20. I often get so carried away by new and exciting things and ideas that I never stop to consider possible complications
21. I tend to change interests frequently

# The 12-item Intolerance of Uncertainty Scale

1= not at all characteristic of me

2= slightly characteristic of me

3= moderately characteristic of me

4= very characteristic of me

5= extremely characteristic of me

1. Unforeseen events upset me greatly

2. It frustrates me not having all the information I need

3. One should always look ahead so as to avoid surprises

4. A small, unforeseen event can spoil everything, even with the best of planning

5. I always want to know what the future has in store for me

6. I can’t stand being taken by surprise

7. I should be able to organize everything in advance

8. Uncertainty keeps me from living a full life

9. When it's time to act, uncertainty paralyzes me

10. When I am uncertain, I can’t function very well

11. The smallest doubt can stop me from acting

12. I must get away from all uncertain situations

# State-Trait Anxiety Inventory: State anxiety subscale

1-not at all

2-a little

3-moderately

4-very much

1. I feel calm (R)

2. I feel secure (R)

3. I am tense

4. I am strained

5. I feel at ease (R)

6. I feel upset

7. I am presently worrying

8. I feel satisfied (R)

9. I feel frightened

10. I feel comfortable (R)

11. I feel self-confident (R)

12. I feel nervous

13. I feel jittery

14. I feel indecisive

15. I am relaxed (R)

16. I feel content (R)

17. I am worried

18. I feel confused

19. I feel steady (R)

20. I feel pleasant (R)

# State-Trait Anxiety Inventory: Trait anxiety subscale

1- Almost never

2- Sometimes

3- Often

4- Almost always

1. I feel pleasant (R)

2. I feel nervous and restless

3. I feel satisfied with myself (R)

4. I wish I could be as happy as others seem to be

5. I feel like a failure

6. I feel rested (R)

7. I am calm, cool, and collected (R)

8. I feel that difficulties are piling up so that I can not overcome them

9. I worry too much over something that really doesn’t matter

10. I am happy (R)

11. I have disturbing thoughts

12. I lack self-confidence

13. I feel secure (R)

14. I make decisions easily (R)

15. I feel inadequate

16. I am content (R)

17. Some unimportant thoughts runs through my mind and bother me

18. I take disappointments so keenly that I can’t put them out of my mind

19. I am a steady person (R)

20. I get in a state of tension or turmoil as I think over my recent concerns and interests.
